# Matrine Restores Colistin Efficacy Against *mcr-1*-Carrying *Escherichia coli*

**DOI:** 10.3390/molecules30102122

**Published:** 2025-05-11

**Authors:** Zhinan Wang, Xiaowei Li, Liang Zhao, Saiwa Liu, Jingjing Du, Xi Jia, Lirui Ge, Jian Xu, Kexin Cui, Yu Ga, Jinxiu Wang, Xi Xia

**Affiliations:** 1 National Key Laboratory of Veterinary Public Health and Safety, College of Veterinary Medicine, China Agricultural University, Beijing 100193, China; wangzn9264@163.com (Z.W.); xiaowei@cau.edu.cn (X.L.); zhaoliang123abc@163.com (L.Z.); liusaiwa0614@163.com (S.L.); djj18731235081@163.com (J.D.); jiax9402@163.com (X.J.); 17614328416@163.com (L.G.); xjian1024@163.com (J.X.); cuikexin0116@163.com (K.C.); gycau1998@163.com (Y.G.); 2 Hainan Provincial Animal Disease Prevention and Control Center, Haikou 571100, China

**Keywords:** matrine, colistin, *mcr-1*, *Escherichia coli*

## Abstract

The emergence of *mcr-1*-mediated colistin resistance has become a critical global health concern, highlighting the urgent need for innovative approaches to restore colistin’s therapeutic potential. In this study, we evaluated the antibacterial activity of four matrine-type alkaloids—namely, matrine, oxymatrine, sophocarpine, and sophoramine—against *mcr-1*-positive *Escherichia coli*. While these alkaloids showed limited efficacy when used alone, the combination of matrine with colistin exhibited remarkable synergistic effects, as demonstrated by checkerboard assays and time-kill curve analyses. The matrine–colistin combination caused minimal erythrocyte damage while effectively attenuating resistance development in vitro. This synergy was further corroborated in a murine infection model, where the combination significantly reduced bacterial loads in target tissues. Mechanistic studies revealed that the matrine–colistin combination enhances antimicrobial activity by disrupting bacterial membrane integrity, increasing intracellular colistin accumulation, and triggering reactive oxygen species-mediated oxidative damage. Collectively, these findings highlight the potential of matrine as a promising adjuvant to overcome colistin resistance, providing a novel therapeutic approach to address the challenge of infections cause by multidrug-resistant Gram-negative bacteria.

## 1. Introduction

With the increasing prevalence of antibiotic resistance, particularly the emergence and spread of carbapenem-resistant *Escherichia coli* (CRE), polymyxins have been reintroduced into clinical practice as a last-resort salvage therapy for infections caused by multidrug-resistant pathogens [1]. However, the extensive use of colistin in both clinical and agricultural settings has led to the global dissemination of the mobile colistin resistance gene, *mcr-1* [2], along with subsequently identified homologs (*mcr-2* through *mcr-10*) [3]. Among these variants, *mcr-1* remains the most prevalent [4], having spread across animal, human, and environmental reservoirs in over 60 countries/regions [5], with a reported prevalence of 5.7% among diarrheal patients in China (2013–2016) [6]. Colistin exerts its bactericidal effect through electrostatic interactions between its cationic residues and the anionic lipid A component of lipopolysaccharide, thereby disrupting membrane integrity [2]. MCR-1 and its variants, which encode phosphoethanolamine transferases, mediate the addition of phosphoethanolamine moieties to lipid A in the outer membrane. This modification neutralizes the negative surface charge and disrupts the electrostatic interactions essential for colistin’s binding and bactericidal activity, leading to acquired resistance [7]. Therefore, there is an urgent need to develop novel strategies to combat *mcr-1*-mediated colistin resistance in pathogens.

Compared to the time-consuming and costly process of traditional antimicrobial development, recent studies have demonstrated that combination therapy, which utilizes two or more therapeutic agents, represents a promising strategy for restoring the efficacy of existing antibiotics and optimizing their clinical use [8]. β-lactamase inhibitors, including clavulanic acid, sulbactam, and tazobactam, have emerged as the most clinically successful adjuvants to date [9]. With an increasing understanding of the pharmacology of natural products, it has become evident that these compounds hold potential for the development of novel antibiotics or antibiotic adjuvants. For instance, three flavonoids—namely, 7,8-dihydroxyflavone, myricetin, and luteolin—have been reported to disrupt bacterial iron homeostasis, thereby enhancing the efficacy of colistin [10]. When co-administered with sub-inhibitory concentrations of colistin, kaempferol-induced iron imbalance results in bacterial cell death [11].

Alkaloids are widely recognized for their antimicrobial properties [12], with many exerting their antimicrobial effects through interactions with bacterial membranes [13]. Chelerythrine has been demonstrated to enhance the efficacy of colistin by impairing bacterial energy production and inhibiting the horizontal transfer of *mcr-1*-bearing plasmids [14]. PA-1, a novel synthetic pyrrolizidine alkaloid, inhibits the growth of *E. coli* and *Staphylococcus aureus* by disrupting their cell membranes [15]. Similarly, berberine exhibits bactericidal activity against *E. coli* and *S. aureus* by compromising their cell membrane integrity, resulting in rapid cation loss in both bacterial species [16]. However, despite these promising findings, colistin adjuvants have not yet been evaluated in human clinical trials due to practical and technical challenges.

*Sophora flavescens*, a traditional Chinese medicinal herb, is widely recognized for its broad spectrum of biological activities [17]. The primary bioactive constituents of its extracts include alkaloids, flavonoids, and other compounds, among which matrine-type alkaloids are particularly notable for their pharmacological properties [18]. Previous studies have shown that the total alkaloids derived from *S. flavescens* have demonstrated significant antibacterial activity [19]. These bioactive matrine-type alkaloids are also characteristic components of *Sophora alopecuroides*, a closely related congeneric species that exhibits similar medicinal properties to *S. flavescens* [20]. Notably, *Sophora alopecuroides* exhibits antibacterial activity against clinically relevant pathogens, including *S. epidermidis*, *E. coli*, and *Helicobacter pylori* [21].

Although matrine-type alkaloids have demonstrated antibacterial efficacy, limited data exist on the activity of four alkaloid monomers—either alone or in combination—against *mcr*-positive *E. coli*, and their bactericidal mechanisms remain unclear. This study evaluated the antimicrobial effects of matrine-type alkaloids, alone or in combination with colistin, against *mcr-1*-positive *E. coli,* with a particular focus on exploring the mechanism behind the synergy of the matrine–colistin combination. Our findings aim to identify novel colistin adjuvants, offering a promising therapeutic strategy against infections caused by drug-resistant Gram-negative bacterial.

## 2. Results and Discussion

### 2.1. Synergistic Activity of Matrine with Colistin

**Figure 1:**
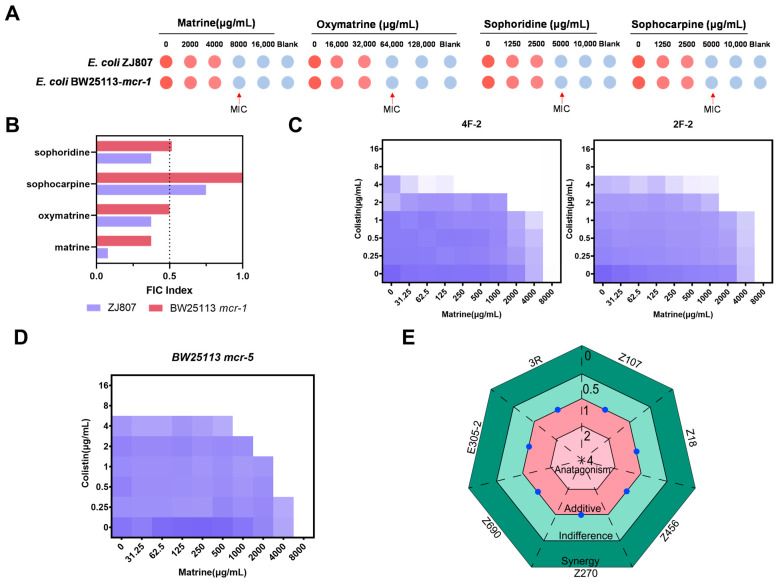
Screening of colistin adjuvants and the synergistic effect of the combination of matrine and colistin. (**A**) Antibacterial activities of matrine-type alkaloids against *E. coli* ZJ807 and BW25113/*mcr-1*. (**B**) Synergistic effects of matrine-type alkaloids with colistin against *E. coli* ZJ807 and BW25113/*mcr-1*. FICI was determined by checkerboard microdilution assays. Synergy was defined as a FICI ≤ 0.5. (**C**) Checkerboard broth microdilution assays of matrine and colistin against wide type *mcr-1*-positive *E. coli*. (**D**) Synergistic effects of matrine and colistin against *mcr-5*-positive *E. coli*. (**E**) Checkerboard broth microdilution assays of matrine with meropenem against carbapenem-resistant *Enterobacteriaceae*.

We investigated the potential bactericidal effects of four major alkaloid monomers derived from *Sophora alopecuroides* extractions that have been reported to exhibit antimicrobial activity. The results indicated that all four alkaloids exhibited modest bactericidal effects against *mcr*-1 positive *E. coli* strains, with a minimum inhibitory concentration (MIC) of 8000 µg/mL for matrine, 64,000 µg/mL for oxymatrine, and 5000 µg/mL for sophoridine and sophocarpine (Figure 1A). We further explored the synergistic effects of the four alkaloids in combination with colistin. Sophocarpine exhibited either indifference or an additive effect when combined with colistin, whereas matrine, oxymatrine, and sophoridine demonstrated synergistic enhancement of antibacterial activity against *mcr-1* positive *E. coli.* Among these, the combination of matrine and colistin showed the highest synergistic activity. This synergistic effect was also observed in other clinically derived *mcr-1*-positive *E. coli* isolates (Figure 1C). We subsequently investigated the synergistic effects of matrine and colistin in other *mcr* variants. As expected, a synergistic effect was observed in *mcr*-*5* carrying *Enterobacteriaceae* (Figure 1D). Given that CRE pose a serious public health threat, we also evaluated the potential synergy between matrine and meropenem against CRE. However, no synergistic effect was observed between matrine and meropenem (Figure 1E). Detailed MIC and fractional inhibitory concentration index (FICI) results are summarized in the Supplementary Materials Table S1.

**Figure 2:**
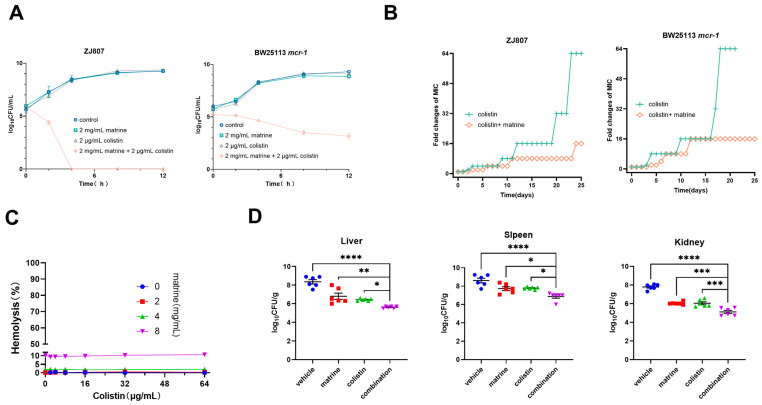
Synergistic antimicrobial activity of matrine and colistin in vitro and in vivo. (**A**) Time-kill assays with colistin (2 μg/mL) in combination with matrine (2000 μg/mL). (**B**) Antibiotic resistance test of colistin in the presence and absence of matrine. (**C**) Hemolytic activity of colistin and matrine at different concentrations in sheep red blood cells. (**D**) Bacterial load in the liver, spleen, and kidney of a mouse model with abdominal infection by *E. coli* ZJ807. (* *p <* 0.05, ** *p <* 0.01, *** *p <* 0.001, **** *p <* 0.0001).

Time-kill curve analysis confirmed the synergy of matrine and colistin. Using colistin at clinical breakpoint concentrations, we observed that, while sub-inhibitory doses of either agent alone (2000 μg/mL matrine or 2 μg/mL colistin) showed limited bactericidal activity, their combination reduced bacterial viability to undetectable levels within 4–12 h (Figure 2A), demonstrating potent in vitro bactericidal activity. Given that the incorporation of antibiotic adjuvants can effectively delay the emergence of bacterial antibiotic resistance [22], we further explored the potential of matrine in preventing colistin resistance. *E. coli* ZJ807 and BW25113/*mcr-1* were serially passaged for 25 days with 1/4 MIC concentration of colistin either alone or in combination with matrine (2000 μg/mL). The colistin-alone group rapidly developed resistance, showing a 32-fold increase in MIC, while resistance increased more slowly in the combination group (Figure 2B). These results suggest that combining matrine with colistin may slow down the increase in colistin MICs.

A critical factor limiting the clinical application of colistin is its potential toxicity, including nephrotoxicity and neurotoxicity [23]. To evaluate whether matrine influences the toxicity profile of colistin, we assessed the hemolytic activity of colistin in the presence of matrine. At a concentration of 8 mg/mL, matrine not only exhibited intrinsic antibacterial activity but also induced the hemolysis of red blood cells (RBCs) (>5%). In contrast, when matrine was combined with colistin at a concentration of 2 mg/mL, the hemolytic activity on RBCs was reduced to 0.2% (Figure 2C), suggesting that the combination had an acceptable hemolytic effect on RBCs.

Given that the combination of colistin and matrine demonstrated excellent synergistic bactericidal activity against active pathogens in vitro, we hypothesized that matrine could reverse MCR-mediated colistin resistance in vivo, and to test this, an *mcr-1*-positive *E. coli* strain (ZJ807) was used to establish a murine peritoneal infection model for evaluating the therapeutic effects of matrine combined with colistin. After 24 h of treatment, both matrine and colistin monotherapies achieved moderate (1–2 log_10_) reductions in bacterial loads compared to the vehicle group. Notably, the colistin–matrine combination therapy demonstrated significantly enhanced efficacy, showing a nearly 2-log_10_ reduction in spleen tissue and an even more pronounced 2–3 log_10_ decrease in both liver and kidney tissues compared to the vehicle control (*p* < 0.0001). Although the combination therapy showed superior bactericidal activity, reducing bacterial loads to levels significantly below those of monotherapies (*p* < 0.05), complete bacterial eradication was not achieved within the 24-h treatment period (Figure 2D). These findings demonstrate that, while the combination of matrine and colistin exerts potent antimicrobial activity in a mouse peritonitis model, further optimization may be required for complete bacterial eradication in this model.

*Sophora flavescens* has been widely used in traditional Chinese medicine, primarily in combination with other medicinal plants in formulations, to treat various conditions, including fever, dysentery, hematochezia, jaundice, oliguria, vulvar swelling, asthma, eczema, inflammatory disorders, ulcers, and diseases related to skin burns [24]. Extracts of *S. alopecuroides* primarily consist of flavonoids, alkaloids, and terpenoids, with alkaloids representing the major bioactive constituents. [16,25]. While the antibacterial activity of the total alkaloids from *S. alopecuroides* has been previously demonstrated [26], the antimicrobial effects of individual alkaloid components remain to be fully elucidated. Matrine, an alkaloid isolated from *S. alopecuroides* [27], belongs to the quinolizidine class and has been reported to exhibit a wide range of pharmacological activities, including antitumor [28], anti-inflammatory [29], antibacterial [30], and antiviral effects [31]. Previous studies indicate that matrine possesses antibacterial activity against *S. aureus* (MIC: 12,500 µg/mL) [26], *E. coli* (MIC ≥ 5120 µg/mL) [32], and *Bacillus subtilis* (MIC: 10,670 µg/mL) [33]. Here, we first investigated the bactericidal effects of four matrine-type alkaloid monomers on *mcr-1*-positive *E. coli*. The results demonstrated that all four alkaloid monomers exhibited bactericidal activity against colistin-resistant *E. coli*, with MIC values ranging from 5000 to 64,000 µg/mL. We further explored the potential of matrine as an antibiotic adjuvant. The results revealed that the combination of colistin and matrine exhibited considerable synergistic effects (FICI ≤ 0.5), with matrine reducing the MICs of colistin to values equal to or below the susceptibility breakpoint (2 μg/mL). Notably, this synergistic effect appears specific to colistin, as matrine only marginally affected meropenem activity (2-fold MIC reduction) against carbapenem-resistant strains. This finding aligns with previous observations that matrine lacks synergistic activity with cefotaxime against extended-spectrum β-lactamase (ESBL)-producing *Klebsiella pneumoniae* strains, suggesting its adjuvant effects may not extend to β-lactam antibiotics in general [34]. Further time-kill studies demonstrated synergistic activity against clinical derived *mcr*-positive strains, while in vivo experiments showed that the combination therapy achieved significant reductions in bacterial loads in drug-resistant infections (*p* < 0.05 compared to monotherapies), though complete bacterial eradication was not attained. Together, these results indicate that matrine specifically enhances colistin activity rather than broadly potentiating other antibiotics in vivo and in vitro.

### 2.2. Matrine Enhances Colistin-Mediated Membrane Damage and Intracellular Drug Accumulation

**Figure 3:**
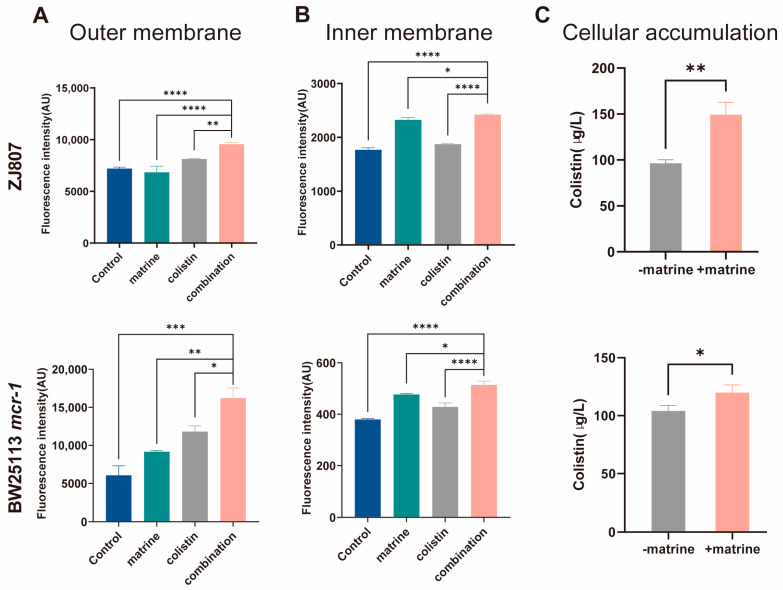
Matrine potentiates colistin-induced damage to bacterial membrane permeability and enhances intracellular colistin accumulation. (**A**) Outer membrane permeability. (**B**) Inner membrane permeability. (**C**) Intracellular colistin accumulation. Data are presented as mean ± SD of three biological replicates. Statistical significance was set at * *p* < 0.05, ** *p* < 0.01, *** *p* < 0.001, and **** *p* < 0.0001.

Having demonstrated that matrine potentiates colistin’s bactericidal activity against resistant pathogens, we next sought to elucidate the underlying mechanisms. Colistin exerts its bactericidal activity primarily by binding to the bacterial outer membrane, selectively targeting lipopolysaccharide, and subsequently disrupting the outer membrane [35]. This prompted us to hypothesize that the mechanism by which matrine reverses colistin resistance involves alterations in bacterial membrane integrity. To test this hypothesis, we first assessed the permeability of both the outer and inner membranes of *E. coli* by measuring the fluorescence intensity of 1-N-phenylnaphthylamine (NPN) and propidium iodide (PI) staining. Our results revealed that treatment with colistin alone induced only a minor increase in membrane permeability. In contrast, the addition of matrine significantly enhanced outer membrane permeability (*p* < 0.01), although matrine alone had little effect on the outer membrane. Notably, matrine alone significantly increased inner membrane permeability (*p* < 0.0001 in ZJ807 and BW25113-*mcr*-1, data not shown in figures) (Figure 3). When combined with colistin, this disruption was further exacerbated. To determine whether matrine enhances the intracellular accumulation of colistin, we employed LC-MS/MS to quantitatively analyze colistin concentrations in *mcr-1*-positive *E. coli* strains. Our results demonstrated a significant increase in intracellular colistin accumulation upon supplementation with matrine (*p* < 0.05).

As the bacterial membrane serves as a critical barrier that protects against external threats and maintains cellular integrity [36], many antibiotics, including colistin, exert their antimicrobial effects by disrupting membrane structures [37,38]. Colistin adjuvants have been shown to induce bacterial death by exacerbating membrane disruption. For instance, melatonin has been demonstrated to restore the membrane-damaging ability of colistin in resistant pathogens [39], chelerythrine enhances the antimicrobial activity of colistin by binding to phospholipids on bacterial membranes and increasing cytoplasmic membrane fluidity [14], and pentamidine potentiates hydrophobic antibiotics through outer membrane disruption [40]. Similarly, matrine also potentiates colistin activity through membrane disruption. Concurrently with membrane disruption, we observed obviously increased intracellular colistin accumulation with matrine treatment (*p < 0.05)*. This phenomenon aligns with previous reports of antibiotic-potentiating mechanisms involving enhanced drug uptake: Vitamin B6 was shown to increase intracellular colistin levels, contributing to its synergistic effect with colistin [41], and MarR inhibitors have been demonstrated to promote colistin accumulation through membrane damage [42].

### 2.3. Colistin-Matrine Combination Promotes Oxidative Damage

**Figure 4:**
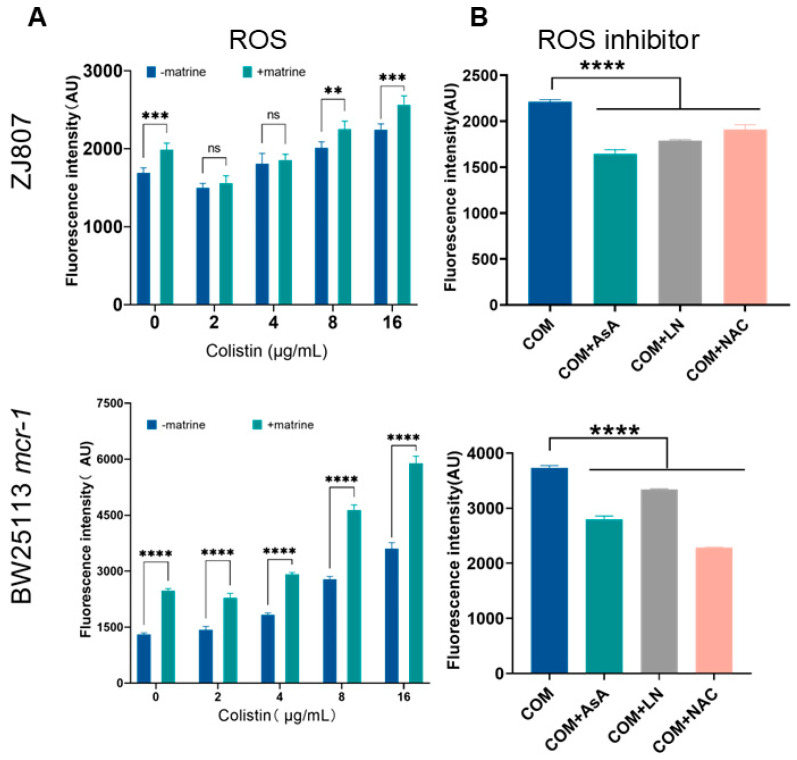
Effects of matrine and colistin on bacterial intracellular ROS levels. (**A**) ROS levels after treatment with colistin in the presence and absence of matrine. (**B**) ROS accumulation with the addition of inhibitors. Data are presented as mean ± SD of three biological replicates. Statistical significance was set at ** *p* < 0.01, *** *p* < 0.001, and **** *p* < 0.0001.

In addition to membrane disruption, colistin induces the rapid killing of Gram-negative bacteria through the production of hydroxyl radicals [11,43]. To further investigate this mechanism, we specifically monitored the accumulation of reactive oxygen species (ROS) in bacterial cells using the fluorescent dye 2′,7′-dichlorodihydrofluorescein diacetate (DCFH-DA), which is oxidized to 2′,7′-dichlorofluorescein (DCF) in the presence of ROS. The results demonstrated that both matrine and colistin alone were capable of inducing ROS production, and high-concentration combination treatment (8/16 μg/mL colistin + 1000 μg/mL matrine) resulted in ROS levels that significantly exceeded those induced by colistin alone (*p* < 0.01) (Figure 4). Furthermore, treatment with various exogenous ROS scavengers, including thiourea, ascorbic acid (AsA), and N-acetyl-L-cysteine (NAC), effectively suppressed this ROS accumulation. These findings suggest that the enhanced ROS production plays a pivotal role in the synergistic antibacterial effect observed between matrine and colistin.

Membrane damage is closely linked to the production of ROS, a key factor in antibiotic-mediated bacterial killing [37]. When membranes are disrupted, the electron transport chain (ETC)—located in the cytoplasmic membrane—becomes impaired, further exacerbating ROS accumulation [35,44]. Given this relationship, oxidative stress serves as a critical indicator of colistin’s antibacterial activity. Notably, gallium nitrate (GaNt) has been reported to enhance intracellular ROS levels in *K. pneumoniae* when combined with colistin, amplifying colistin’s lethality against wild-type cells [44]. This aligns with studies showing that membrane-targeting antimicrobial peptides can trigger ROS production as part of their bactericidal mechanism [45]. Our study demonstrates that matrine significantly potentiates ROS accumulation in combination with colistin, suggesting a similar oxidative stress-driven enhancement of antibacterial activity.

## 3. Materials and Methods

### 3.1. Bacteria and Reagents

The strains used in this study are listed in Table 1. The *mcr-1* bearing plasmid IncI2-*mcr-1* was extracted from ZJ807 and subsequently transformed into *E. coli* BW25113, resulting in the strain BW25113::IncI2-*mcr-1* [14]. Matrine, oxymatrine, sophocarpine, and sophoramine were obtained from Chengdu Herbpurify Co., LTD (Sichuan, Beijing). Other chemical reagents, including colistin, meropenem, ascorbic acid, N-acetyl-L-cysteine, thiourea, and fluorescent probes (1-N-phenylnaphthylamine, propidium iodide, and 2′,7′-dichlorofluorescin diacetate) were purchased from Shanghai Aladdin Biochemical Technology Co., Ltd. (Shanghai, China).Luria-Bertani (LB) agar, LB broth, and Mueller-Hinton Broth (MHB) were purchased from Beijing LanBridge technology CO.,LTD (Beijing, China). Phosphate-Buffered Saline (PBS) and N’-a-hydroxythylpiperazine-N’-ethanesulfanic acid (HEPES) buffer were acquired from Beijing Solarbio Technology CO., Ltd. (Beijing, China).

### 3.2. MIC Assay and Antimicrobial Combination Test

The standard broth microdilution method was employed to determine the MICs of four alkaloids (matrine, oxymatrine, sophocarpine, and sophoridine) against *mcr*-positive *E. coli* strains ZJ807 and BW25113-*mcr*-1 following Clinical and Laboratory Standards Institute (CLSI) guidelines (documents M07 and M100). The initial concentrations of the test compounds (32,000 μg/mL for matrine, 256,000 μg/mL for oxymatrine, 20,000 μg/mL for sophocarpine, and 20,000 μg/mL for sophoridine) were loaded into the first well of a 96-well plate and serially diluted two-fold. Bacterial suspensions (adjusted to 1 × 10⁶ CFU/mL in MHB) were then added to each well. After 18 h of static incubation at 37 °C in the dark, MICs were defined as the lowest drug concentrations that completely inhibited visible bacterial growth. All determinations were performed with three biological replicates, each consisting of three technical replicates.

Checkerboard assays were conducted first to explore the interactions between colistin and four alkaloids (matrine, oxymatrine, sophocarpine, and sophoramine) against *mcr*-1-positive *E. coli* strains ZJ807 and BW25113-*mcr*-1. Next, the synergistic potential of matrine–colistin combinations was assessed against additional *mcr*-positive isolates, including BW25113-*mcr*-5, 2F-2, and 4F-2. Finally, matrine–meropenem combinations were examined against ESBL-producing *Enterobacteriaceae* to determine whether matrine could restore meropenem susceptibility. In brief, 100 μL MHB was dispensed into each well of a 96-well plate. Alkaloids and antibiotics were then serially diluted twofold along the vertical and horizontal lines, respectively, with the first well containing a final concentration of 2 × MIC for both compounds. A total of 100 μL overnight-grown bacterial suspensions were added to achieve a final concentration of approximately 1 × 10^6^ colony-forming units (CFU) per millilitre, followed by incubation at 37 °C for 18 h. To quantify the level of interactions between alkaloids and antibiotics, the FICI was calculated as follows:

FICI = MIC_ab_/MIC_a_ + MIC_ba_/MIC_b_

where MIC_a_ and MIC_b_ represent the individual MIC values of compound A and compound B, respectively; MIC_ab_ is the MIC of compound A in combination with compound B; MIC_ba_ is the MIC of compound B in combination with compound A. Synergy was defined as an FICI ≤ 0.5, while an FICI between 0.5 and 1 indicated additive, and an FICI between 1 and 2 indicated indifferences. An FICI > 2 was interpreted as antagonism.

### 3.3. Time-Kill Curves

ZJ807 and BW25113/*mcr-1* were cultured overnight in MHB and adjusted to match a 0.5 McFarland turbidity. A 1:100 dilution of bacterial suspension was then treated with either 2 μg/mL colistin alone, 2000 μg/mL matrine alone, or a combination of both agents. During a 12-h incubation at 37 °C with shaking at 260 rpm, 100 μL aliquots of the culture were collected at 0, 2, 4, 8, and 12 h. Each aliquot was serially diluted in PBS through a 10-fold gradient (10^0^ to 10⁶) and plated on LB agar. The plates were incubated overnight, and the colonies were counted to construct the time-kill curves.

### 3.4. Resistance Development Assay

Serial passage experiments were conducted to assess colistin resistance development in the absence or presence of matrine. ZJ807 and BW25113-*mcr-1* in the exponential phase were diluted 1:1000 into fresh MHB supplemented with 2000 μg/mL matrine and 4, 8, and 16 μg/mL colistin, respectively. The 2000 μg/mL matrine concentration was chosen based on the critical role of antibiotic concentration in shaping antimicrobial resistance gene dynamics, which directly drive resistance evolution [49,50]. After culturing for 24 h at 37 °C with shaking at 260 rpm, the highest concentration showing visible bacterial growth was selected for MIC determination. Meanwhile, the selected bacterial cells were diluted 1000-fold in fresh MHB supplemented with 2000 μg/mL matrine and colistin (at half, equal, and double the maximum concentration used in the previous passages) for subsequent passages. This process was repeated for 25 days, and colistin was used as the control.

### 3.5. Assessment of Matrine on Colistin-Induced Hemolysis

To evaluate whether matrine modulates the toxicity of colistin, we assessed the hemolytic activity of colistin in the presence of matrine based on a previously reported method [51]. Fresh defibrinated sheep blood was centrifuged, and the resulting pellet was resuspended in PBS to prepare an 8% sheep RBC suspension. Biologically relevant concentrations of matrine (0–16 mg/mL) and colistin (0–128 μg/mL) were selected to incubate with an equal volume of the 8% RBC suspension at 37 °C for 1 h. PBS and ultrapure water were used as the negative control and positive control, respectively. After incubation, the absorbance of released hemoglobin was measured at 576 nm to calculate the hemolysis rate. The percentage of hemolysis was determined using the following formula: hemolysis percentage = [OD576_sample_−OD576_PBS_]/[[OD576_water_ − OD576_PBS_] × 100%.

### 3.6. Mouse Infection Assays

Female BALB/C mice aged 6–8 weeks (weighing 18.0–20.0 g) were purchased from Beijing Vital River Laboratory Animal Technology Co., Ltd. Prior to the experiments, all mice were acclimatized for 7 days under controlled conditions, including a 12-h light/dark cycle and a temperature of 23 ± 2 °C. To establish a peritonitis–sepsis infection model, mice were intraperitoneally inoculated with 1 × 10^9^ CFUs of *E. coli* ZJ807 per mouse. Within 24 h post-infection, mice developed characteristic clinical signs including hypothermia, tremors, diarrhea, and septic shock, with subsequent necropsy revealing peritoneal hyperemia and intestinal wall congestion. The model exhibited >90% mortality within 7 days, and bacterial cultures confirmed *E. coli* ZJ807 recovery from organ homogenates, collectively validating successful infection establishment.

At 1 h post-infection, mice were intraperitoneally administered PBS, 5 mg/kg colistin, 40 mg/kg matrine, or a combination of 5 mg/kg colistin and 40 mg/kg matrine (*n* = 6 per group). The doses of matrine (40 mg/kg) and colistin (5 mg/kg) were carefully selected to balance efficacy and safety. Specifically, matrine’s dose was set fourfold lower than that of murine LD_50_ (157 mg/kg) to ensure safety, whereas colistin’s does was minimized to reduce nephrotoxicity while preserving antimicrobial activity. After 24 h of treatment, the mice were euthanized by cervical dislocation, and the bacterial load in the liver, spleen, and kidney were quantified by serial dilution plating of organ homogenates. All animal experiments were approved by the Committee on Animal Welfare and Ethics of China Agricultural University (AW82113202-2-1).

### 3.7. Outer Membrane Integrity

The permeability of *E. coli* ZJ807 and BW25113-*mcr-1* were assessed using the fluorescent probe NPN as previously described [47]. Briefly, ZJ807 and BW25113-*mcr-1* cells at the mid-logarithmic phase were harvested and diluted with a PBS buffer to OD_600_ of 0.5. Non-lethal final concentrations of 1000 μg/mL matrine and 16 μg/mL colistin were used to treat bacteria for 30 min at 37 °C with shaking at 260 rpm to ensure cell viability during antimicrobial effect evaluation. Subsequently, the cells were pelleted by centrifugation at 7000 rpm for 6 min and resuspended in an HEPES buffer containing 10 μM NPN. After 30 min of incubation in the dark, fluorescence intensity was measured at an emission wavelength of 420 nm following excitation at 355 nm.

### 3.8. Inner Membrane Integrity

For inner membrane integrity assessment using PI, the same sub-lethal concentrations (1000 μg/mL matrine and 16 μg/mL colistin) as those used in the NPN uptake assay were maintained. Briefly, bacteria at the mid-logarithmic phase were resuspended in PBS containing 10 μM PI and incubated in the dark for 30 min. To remove unbound PI, the cells were pelleted by centrifugation (7000 rpm, 6 min) and washed three times with PBS. The washed cells were then resuspended in PBS containing 1000 μg/mL matrine and 16 μg/mL colistin. After incubation for 30 min, fluorescence intensity was measured at an excitation wavelength of 535 nm and an emission wavelength of 615 nm.

### 3.9. Intracellular Colistin Accumulation

Bacteria at the mid-logarithmic phase were harvested and diluted in PBS. Following treatment with 1000 μg/mL matrine and 16 μg/mL colistin for 30 min—maintaining the same sub-lethal concentrations used in our NPN uptake and PI permeability assays—the bacterial pellets were washed twice with PBS and resuspended in a 30% methanol/water solution. The suspensions were vortexed thoroughly and subjected to cell lysis using ice-bath ultrasonication. Subsequently, the samples were centrifuged at 21,000 rpm for 20 min, and the resulting supernatants were collected and diluted tenfold with 30% methanol/water for further analysis. The samples were filtered through a 0.22 μm filter and injected into a UHPLC-MS/MS system (Waters, TQ-XS). The instrument parameters are detailed in the Supplementary Materials.

### 3.10. Total ROS

The levels of ROS in *E. coli* strains ZJ807 and BW25113/*mcr-1* treated with matrine and colistin were measured using DCFH-DA. Briefly, overnight cultures were diluted 1:100 in fresh LB broth and grown to the exponential phase. The cells were harvested by centrifugation at 7000 rpm for 6 min and resuspended in sterile PBS buffer to an OD_600_ of 0.5. Subsequently, bacterial cultures were incubated with matrine (1000 μg/mL) and colistin (2–16 μg/mL) for 30 min at 37 °C (260 rpm) under gradient non-bactericidal conditions. After treatment, the bacteria were collected by centrifugation and resuspended in PBS containing 10 μM DCFH-DA. The solution was incubated in the dark for 30 min. Fluorescence intensity was measured at an excitation wavelength of 488 nm and an emission wavelength of 525 nm.

To evaluate the role of ROS in antibacterial activity, we assessed ROS accumulation in the presence of three established ROS inhibitors: AsA, NAC, and thiourea. Bacterial suspensions were co-incubated with 50 mM inhibitor in combination with 1000 μg/mL matrine and 16 μg/mL colistin for 30 min. Cells were harvested by centrifugation at 7000 rpm for 6 min, washed twice with PBS, and resuspended in PBS containing 10 μM DCFH-DA. After 30 min of dark incubation at 37 °C, fluorescence intensity was measured at excitation and emission wavelengths of 488 nm and 525 nm, respectively.

### 3.11. Statistical Analyses

All statistical analyses were performed using GraphPad Prism 9.1, with data presented as mean ± standard deviation. Normality was assessed using Shapiro–Wilk tests (α = 0.05). For normally distributed data, two-group comparisons were analyzed with independent samples t-tests after confirming the homogeneity of variance with Brown–Forsythe tests (*p* > 0.05), while multi-group comparisons employed either one-way ANOVA with Tukey’s post-hoc test when variances were equal or Welch’s ANOVA with Dunnett’s T3 post-hoc test when variances were unequal (Brown–Forsy, *p* ≤ 0.05). Non-normally distributed data were analyzed using the Kruskal–Wallis test followed by Dunn’s multiple comparisons test. For comparisons between colistin and colistin + matrine treatments, two-way ANOVA was performed when both normality and homoscedasticity assumptions were met, with Tukey’s post-hoc test for pairwise comparisons. All analyses maintained a significance threshold of α = 0.05.

## 4. Conclusions

In this study, we demonstrated that matrine, a natural alkaloid derived from *Sophora alopecuroides*, significantly potentiates the antibacterial activity of colistin against *mcr-1*-positive Gram-negative *E. coli*. Our findings revealed that the combination of matrine and colistin exhibits potent synergistic effects in both in vitro and in vivo models. Matrine enhances colistin’s efficacy by disrupting bacterial membrane integrity, increasing intracellular colistin accumulation, and promoting ROS-mediated oxidative damage. These results highlight the potential of matrine as a promising antibiotic adjuvant to combat infections caused by colistin-resistant pathogens, offering a novel strategy to address the growing threat of multidrug-resistant Gram-negative bacteria.

## Figures and Tables

**Table 1:** Strains of *E. coli* used in this study.

Strain ID	Origin (Isolation Location)	Resistance Profile	MIC (μg/mL)	Reference
ZJ807	Zhejiang, clinical	*mcr-1*	Colistin: 4	[46]
BW25113	Lab-constructed	*mcr-1*	Colistin: 4	[14]
4F-2	Unknown	*mcr-1*	Colistin: 4	this study
2F-2	Unknown	*mcr-1*	Colistin: 4	[47]
BW25113	Lab-constructed	*mcr-5*	Colistin: 4	this study
3R	Shandong, environmental	*bla*_NDM-5_	Meropenem: 128	[48]
Z107	Zhejiang, clinical	*bla*_OXA-232_	Meropenem: 32	this study
Z18	Zhejiang, clinical	*bla*_NDM-5_	Meropenem: 32	this study
Z456	Zhejiang, clinical	*bla*_KPC-2_	Meropenem: 32	this study
Z270	Zhejiang, clinical	*bla*_NDM-4_	Meropenem: 32	this study
Z690	Zhejiang, clinical	*bla*_NDM-5,_ *bla*_KPC-2_	Meropenem: 64	this study
E305-2	Zhejiang, clinical	*bla*_IMP-4_	Meropenem: 8	this study

## Data Availability

The data that support the findings of this study are available from the corresponding author (X.X.) upon reasonable request.

## Supplementary Materials

The following supporting information can be downloaded at: https://www.mdpi.com/article/10.3390/molecules30102122/s1, Table S1. Synergistic activity of antibiotics combined with matrine-type alkaloids against *E. coli;* Table S2. MS parameters for colistin.
