# Supplementary material for: Matrine Restores Colistin Efficacy Against mcr-1-Carrying Escherichia coli
Source: Molecules. 2025 May 11;30(10):2122. doi: 10.3390/molecules30102122 (PMC12113751; doi:10.3390/molecules30102122)
Supplement: Supplementary file 1 [file molecules-30-02122-s001.zip › molecules-3562458-supplementary.pdf]

Supplementary materials

Table S1. Synergistic activity of antibiotics combined with matrine-type alkaloids against *E. coli*

**Bacterial strain**	**Antibiotic MIC (μg/mL)**			**Adjuvant MIC (μg/mL)**			**FICI**	**Outcome**
	Antibiotic	Alone	Combined	Agent	Alone	Combined		
**ZJ807**	colistin	4	0.25	Matrine	8000	125	0.078125	Synergistic
	colistin	4	0.5	Oxymatrine	64,000	16000	0.375	Synergistic
	colistin	4	0.5	Sophoridine	5000	1250	0.375	Synergistic
	colistin	4	2	Sophocarpine	5000	1250	0.75	Indifference
**BW25113-*mcr-1***	colistin	4	1	Matrine	8000	1000	0.375	Synergistic
	colistin	4	1	Oxymatrine	64,000	16000	0.5	Synergistic
	colistin	4	2	Sophoridine	5000	78.125	0.516	Synergistic
	colistin	4	2	Sophocarpine	5000	2500	1	Additive
**4F-2**	colistin	8	2	Matrine	8000	250	0.28125	Additive
**2F-2**	colistin	8	2	Matrine	8000	2000	0.5	Additive
**BW25113-*mcr-5***	colistin	8	0.5	Matrine	4000	500	0.1875	Additive
**3R**	meropenem	128	64	Matrine	8000	4000	1	Additive
**Z107**	meropenem	32	16	Matrine	8000	4000	1	Additive
**Z18**	meropenem	32	16	Matrine	8000	4000	1	Additive
**Z456**	meropenem	32	16	Matrine	8000	4000	1	Additive
**Z270**	meropenem	32	16	Matrine	8000	4000	1	Additive
**Z690**	meropenem	64	32	Matrine	8000	4000	1	Additive
**E305-2**	meropenem	8	4	Matrine	8000	4000	1	Additive

Chromatography and mass spectrometry conditions for colistin quantification

Samples were analyzed using Waters Xevo TQ-XS triple quadrupole mass spectrometer coupled with an I-Class Plus Acquity UPLC system. Chromatographic separation was performed on an ACQUITY UPLC BEH C_{18} Column (Waters, 2.1 × 50 mm, 1.7 µm) maintained at 40 °C. The injection volume was 5 µL, with a flow rate of 0.4 mL/min. The mobile phases consisted of solvent A (0.5% formic acid in water) and solvent B (0.5% formic acid in acetonitrile). The linear gradient elution was programmed as follows: 0–0.5 min: 95% A; 0.5–2 min: 95% to 30% A; 2–3 min: 30% A; 3–3.1 min: 30% to 5% A; 3.1–4 min: 5% A; 4–4.5 min: 5% to 95% A; 4.5–5.5 min: 95% A.

Detection of target compounds was conducted using electrospray ionization in positive mode (ESI+), operating in multiple reaction monitoring (MRM) mode. The capillary voltage was set at 2.8 kV. The desolvation and source temperatures were maintained at 500 °C and 150 °C, respectively. Gas flows were set as follows: desolvation gas at 800 L/h and cone gas at 150 L/h. The transitions and corresponding parameters for colistin are detailed in Table S2.

Table S2. MS parameters for colistin

Compound	Precursor ion (*m/z*)	Product ion (*m/z*)	Cone voltage（V）	Collision energy（eV）
Colistin-A	390.71>101.12	384.8 / 101.1 [a]	44	16 / 8
Colistin-B	385.90>101.12	380.1 / 101.1 [a]	52	16 / 8

[a] Transitions for quantification.
